# A Clinical Investigation on the Theragnostic Effect of MicroRNA Biomarkers for Survival Outcome in Cervical Cancer: A PRISMA-P Compliant Protocol for Systematic Review and Comprehensive Meta-Analysis

**DOI:** 10.3390/genes13030463

**Published:** 2022-03-05

**Authors:** Peter Shaw, Raghul Senthilnathan, Srivarshini Sankar, Ilamathi Ilangovan, Gothandam Kodiveri Muthukaliannan, Siddhartha Baxi, Ravishankar Ram Mani, Mogana Rajagopal, Sasikala Chinnappan, Ashok Kumar Balaraman, Deepa Suresh, Sunil Krishnan, Madanmohan Gupta, Thangavel Muthusamy, Chitraabaanu Paranjothy, Rama Jayaraj

**Affiliations:** 1 Department of Artificial Intelligence, Nanjing University of Information Science and Technology (NUIST), Nanjing 210044, China; 100001@nuist.edu.cn; 2 Menzies School of Health Research, Darwin 0810, Australia; 3 School of Bio Sciences and Technology, Vellore Institute of Technology (VIT), Vellore 632014, Tamil Nadu, India; raghulsnn@gmail.com (R.S.); srivarshini307@gmail.com (S.S.); ilangovanilamathi@gmail.com (I.I.); gothandam@gmail.com (G.K.M.); 4 GenesisCare Gold Coast Radiation Oncologist, John Flynn Hospital, 42 Inland Drive, Tugun 4224, Australia; Siddhartha.baxi@genesiscare.com; 5 Department of Pharmaceutical Biology, Faculty of Pharmaceutical Sciences, UCSI University Kuala Lumpur (South Wing), No.1, Jalan Menara Gading, UCSI Heights, Kuala Lumpur 56000, Malaysia; Ravishankar@ucsiuniversity.edu.my (R.R.M.); mogana@ucsiuniversity.edu.my (M.R.); sasikala@ucsiuniversity.edu.my (S.C.); ashokkumar@ucsiuniversity.edu.my (A.K.B.); 6 Division of Endocrinology, Department of Internal Medicine, Mayo Clinic Florida, Jacksonville, FL 32224, USA; deepa.suresh@mayo.edu; 7 Department of Radiation Oncology, Mayo Clinic Florida, 4500 San Pablo Road S, Jacksonville, FL 32224, USA; Krishnan.Sunil@mayo.edu; 8 School of Pharmacy, Faculty of Medical Sciences, The University of the West Indies, St. Augustine 3303, Trinidad and Tobago; madanmohan.gupta@sta.uwi.edu; 9 Research and Development, Sree Balaji Medical College and Hospital, Bharath Institute of Higher Education and Research, 7 CLC Works Road, Chromepet, Chennai 600044, Tamil Nadu, India; thangavelmuthusamy.research@bharathuniv.ac.in; 10 Department of Public Health, University of Essex, Leeds LS1 2RP, UK; cp20010@essex.ac.uk; 11 Clinical Sciences, Northern Territory Institute of Research and Training, Darwin 0909, Australia

**Keywords:** cervical cancer, miRNA, chemoresistance, biomarkers

## Abstract

Background: The most often diagnosed malignancy in women worldwide is cancer of the cervix. It is also the most prevalent kind of gynecological cancer in women. This cancer originates in the opening of the cervix and spreads through sexual contact. Even though human papillomavirus (HPV) may not cause cancer immediately, it does develop over time as a result of the virus’s lengthy persistence to cause dysplastic changes overtime, particularly in high-risk kinds. The primary objective of this research is to see if miRNAs are dysregulated as a result of treatment resistance in cervical cancer (CC). The aim is to see if these microRNAs may be utilized as biomarkers for detecting chemoresistance in CC, particularly for clinical applications. Methods: The recommended protocol for comprehensive study and meta-analysis (PRISMA-P) standards will be utilized for the analysis and data interpretation. The bibliographic databases will be methodically searched using a combination of search keywords. Based on established inclusion and exclusion criteria, the acquired findings will be reviewed, and data retrieved from the selected scientific papers for systematic review. We will then construct a forest from the pooled Hazard ratio (HR) and 95% C.I. values, data obtained using the random-effects model. Discussion: The focus of this study is to identify the function of miRNAs as a chemoresistance regulator and determine if they have the potential scope to be considered as biomarkers for cervical cancer. Through this systematic review and meta-analysis, the goal is to collect, compare, and analyze the data pertaining to the role of miRNAs in cervical cancer, thereby, enabling us to understand the role they play in chemosensitivity.

## 1. Introduction

### 1.1. Background

Cervical cancer (CC) is one among the most reported cancers in women [1]. It is also reported to be the most frequent type of gynecological cancer in women [2]. The squamous cell cervical carcinoma makes up 75% of the cases, whereas 25% are adenocarcinomas [3,4]. It was observed that most of those suffering from this malignancy are victims of Human Papilloma Virus (HPV) [5]. This cancer develops in the cervical opening and spreads as a result of sexual contact. Although HPV does not cause immediate cancer formation, it manifests over time due to the prolonged presence of the virus, especially the high-risk subtypes [5,6,7].

The HPV infects either the basal skin cells, specifically the epithelial cells or the inner lining of tissues. Based on their ability to develop CC, they are categorized as high risk and low risk. Low-risk HPV variations include serotypes 6, 11, 42, 43, and 44, whereas high-risk HPV variants include 16, 18, 31, 33, 34, 35, 39, 45, 51, 52, 56, 58, 59, 66, 68, and 70 [8,9]. Among these, serotypes 16 and 18 are the most frequently diagnosed CC and are responsible for 70% of the total cases [6,10,11,12,13,14]. HPV is a very widespread with over 14,000,000 individuals annually infected with this sexually transmitted disease worldwide [15]. The link among HPV as well as CC has become so profound that it surpasses the link among smoking and lung cancer [16]. Apart from HPV, sexually transmitted diseases such as chlamydia, Human Immunodeficiency Virus (HIV) and Herpes Simplex Virus (HSV) are also causative agents of this type of cancer. Other possible causes of cervical cancer include multiple sexual partners, parity, consumption of oral contraceptive pills, smoking, obesity, and dietary habits [4,17,18].

There are a variety of symptoms during the manifestation of cervical cancer. During the early-stage slight blood spotting could be present between the menstrual period cycles; post-menopausal bleeding can also occur. There can also be an increase in vaginal discharge accompanied by a bad smell and bleeding after sexual intercourse. In extreme conditions, back, leg, or pelvic pain can become persistent. Other symptoms such as loss of weight, fatigue, loss of appetite, and swelling of lower extremities can be observed [19].

CC is mostly spread in underdeveloped and developing countries compared to developed countries, due to the lack of effective screening, cancer treatment facilities, and early detection programs [20]. Usually, in countries with higher incomes, girls and women are vaccinated and screened regularly against HPV. Hence, the incidence rate is lower, and the cancer is treatable. In third world countries, due to limitations, unavailability of proper health care and awareness, the incidence and mortality rates are higher in these countries. Indeed, it is the leading cause of mortality in 38 low-income countries, primarily in South America and Sub-Saharan Africa [21]. By the time it is identified, it becomes too late to treat. Therefore, irrespective of the treatability of the disease, it is in the fourth most detected cancers worldwide among females [11,19,22].

Various strategies such as Global Alliance for immunization and vaccination, single visit and see treat approaches, and high-risk HPV testing have been introduced to reduce the impact of this cancer type, specifically in underdeveloped and developing nations [20]. To curb this issue, WHO has also released a worldwide strategy to eradicate CC as a public health issue on 17 November 2017. All the 194 WHO abiding countries have taken part in this initiative to spread awareness and control the spread of this cancer [12,23]. WHO devised a consortium for the elimination of cervical cancer to spread its strategy for CC eradication. The results from the former and the comparative modeling analysis carried out by Marc, Brisson et al., 2020, foresee that this cancer could become extinct in all countries by 2021 [24]. The comparative modeling analysis for mortality also predicted that 74 million cases and 62 million deaths can be prevented through the enforcement of WHO’s strategy [25].

miRNAs have been identified to have important roles in cancer resistance to medicines and treatments. This is supposed to happen when miRNAs deactivate numerous pathways that are critical for cancer suppression, such as apoptosis and DNA repair. Many miRNAs have recently been discovered to have important roles in chemoresistance to cervical cancer, either through overexpression of the individual miRNA or by reduced expression dependent on their target genes [26,27]. Similarly, they can either raise or decrease the level of resistance to the chemotherapy, thereby playing a dual role [28,29]. Apparently, treatments can affect changes in the expression levels of miRNA, imparting resistance to some medications [27]. As a result, research is being performed to increase chemosensitivity by examining the change in miRNA expression while administering chemo medications [30].

### 1.2. Epidemiology

Over the years, there has been an increase in the number of infected females from 10 to 40% making CC amongst the topmost death causing cancers. Around 99% of the CCs is a result of HPV infections [19]. In 2012, there were 528,000 new cases and 266,000 fatalities, but in 2018, there were 528,000 new cases and 266,000 deaths. Data collected estimated around 570,000 cases and 311,000 deaths due to CC [2,31]. The 2020 report revealed that 604,237 women were diagnosed, while 342,000 deaths occurred worldwide. This constitutes about 6.5% of the total female population globally [32].

Cervical cancer is the most common kind of cancer in 28 countries and a leading cause of death in 42. Most of these countries are located in Sub-Saharan Africa and Southeast Asia [22,33]. China and India alone constitute 1/3rd of the total cervical cancer cases [12]. Every year, around 120,000 women in India are diagnosed with cervical carcinoma, accounting for 15.2% of all cervical cancer fatalities globally [34]. Countries situated in various parts of Africa account for more than 30 per 100,000 cases [35].

Further data revealed that North America and Western Europe have 6.6 and 7.3 cases for every 100,000 individuals. In countries such as sub-Saharan Africa, Latin America, and Caribbean regions, there are 34.8 and 21.2 cases for every 100,000 people, respectively [36]. Around 85% of the deaths from this cervical carcinoma were among populations from underdeveloped or developing nations because of poor population-based screening and vaccine coverage [2,21]. According to reports up to May 2020, only about a third of low- and middle-income countries have implemented HPV vaccination programs, compared to 80% in developed nations [32]. The death rate was estimated to be 18 times higher in these countries compared to the more affluent nations [11]. It is predicted that, by 2030, almost all cervical cancer cases will be just from low- and middle-income countries [21,35].

### 1.3. Objectives

1. To determine which miRNAs are dysregulated due to chemoresistance in cervical cancer patients.
2. To examine the overall effect of miRNA expression on chemoresistance in patients with cervical cancer.
3. To determine dysregulated miRNAs and their role as biomarkers to detect chemotherapy resistance in cervical cancer.

### 1.4. Review Questions

1. What are the miRNAs that are highly expressed in cervical cancer?
2. Which miRNAs are dysregulated in cervical cancer leading to chemoresistance?
3. Which miRNAs could be used as a biomarker in the detection of chemoresistance in cervical cancer?

### 1.5. Rationale

#### 1.5.1. The Importance of This Study

miRNAs are non-coding RNAs that generally have a short life and gene expression altering capabilities. They seem to have a role in cervical carcinogenesis. Many articles covering the topic of CC discuss a single type of miRNA and its specific role [6,37,38,39,40,41]. They elaborate on how the respective miRNA causes resistance or sensitivity and the mechanism involved with decrease or increase in the chemoresistance of a specific anti-cancer drug. There is a lack of a research that focuses on all the miRNAs.

#### 1.5.2. How Will the Study Address This Issue?

By utilizing systematic review and meta-analysis, our study will provide a consolidated data set, thus allowing us to connect this data to clinical practice. Our study will focus on how the various miRNAs can be used as a biomarker to detect the resistance provided by multiple drugs. This study will also analyze how these biomarkers can be incorporated therapeutically as a chemoresistance regulator in cervical cancer.

## 2. Materials and Methods

This research will concentrate on miRNAs and their involvement in the regulation chemoresistance in CC. The PRISMA (Preferred Reporting Items for Systematic Review and Meta-Analyses) recommendations will be followed and adhered to in this study’s methodology.

### 2.1. Search Methods

To obtain the needed data, we will consider several types of miRNAs with known drug resistance. This includes both positive and negative correlations of the miRNAs with cervical cancer. It is necessary to apply the correct formal strategy to collect the relevant research. To gather the relevant papers, the SCOPUS, EMBASE, Science Direct, Medline, PubMed, and Google Scholar reference databases will be used. The search strategy will use relative key terms; these are mentioned in Table 1. The acquisition of data will not be limited to a specific place and will be collected irrespective of the ethnicity of those suffering from this cancer. The primary stage of screening will concentrate on the title and abstract of the study. The following stage will focus on conducting a thorough investigation of each of the chosen studies to obtain the necessary and valid information. Only the full-text, open-access articles will be included in this study. To integrate this qualifying research, we will retrieve the required articles from the bibliographies of the screened publications. If there are any disagreements, they will be addressed by an impartial third party.

In order of significance, the following are the potential keywords to be used in this search strategy employing Medical Subject Headings (MeSH) to build the core search: miRNA, Cervical cancer, Chemoresistance, Upregulation and downregulation of miRNA expression, as well as deregulation and dysregulation, Biomarkers, Treatment (Resection surgery, Radiotherapy, Chemotherapy), Clinical study and miRNA expression on chemoresistance in patients with CC.

### 2.2. Study Selection

The studies will be chosen at first based on two contributors’ individual judgments after reading the titles and abstracts of the articles. In both retrospective cohort and prospective cohort studies, miRNA expression in cervical cancer patients will be evaluated in relation to the expression of miRNAs against a variety of medications used to treat cervical cancer. Studies having study-specific data (e.g., hazard ratios, 95% confidence intervals) or sufficient data to generate outcome data will be rigorously examined. The study setting will not be a constraint. After the relevant articles have been screened in, two contributors will individually undertake a detailed study of the full-text articles using the previously stated selection criteria. Any dispute between the two contributors will be handled through conversation. A collaborative consensus or an independent evaluator will resolve any significant discrepancies. A flow chart explaining this method will be prepared to aid transparency. Following the selection of suitable articles, they will be further evaluated using the set of criteria. For the systematic review, only those that fulfilled the inclusion criteria will be considered.

### 2.3. Guidelines for Considering Studies for Review

The basis for determining which papers to include in the review is broad and comprehensive. Every medical trial that investigates miRNA expression in individual participants or fresh as well as preserved tissue samples and links it with chemoresistance will be analyzed in this systematic review and meta-analysis. A complete criterion is needed to overcome the difficulty caused by the limited body of published articles on the subject, as this limitation is often a significant hurdle in systematic reviews and meta-analyses.

#### 2.3.1. Inclusion Criteria

- Foremost criteria will focus on collecting articles pertaining to miRNAs and their regulatory role in CC drug resistance.
- Other inclusions include:
- Studies relating to the action of miRNAs against a variety of drugs used for treating CC.
- Different miRNAs’ mechanisms of action on cancer causing cervical cells.
- Studies pertaining to both chemo-sensitive and chemoresistant modulation by miRNAs on CC cells.
- Studies that follow the PRISMA guidelines and may therefore be utilized in systematic reviews and meta-analyses.

#### 2.3.2. Exclusion Criteria

- Articles written in other languages, not English.
- Letter to editors, review articles, case studies, non-human studies.
- Unpublished articles, abstruse data, theses, and conference/seminar presentations.
- Studies without full accessibility.
- Duplicate studies.

#### 2.3.3. Data Collection and Management

Primarily, the writers’ data will be saved in separate Microsoft Excel spreadsheets. The Excel sheets would be pooled and duplicates will be removed after all the data has been collected. The findings of the search will be included in the most recent report, as well as depicted in a PRISMA workflow diagram and model based on PRISMA regulations.

EndNote will be utilized to post all recognized citations after the data has been gathered, and duplicates will be eliminated. After that, the journal articles will be analyzed, and the following information will be gathered: Information about the authors, Publication year, The location of the investigation, Patient information (Age, Gender, Ethnicity), CC risk profile (HPV (human papillomavirus infection, sexual history, smoking, multiple full-term pregnancies, chlamydia infection), and Mortality.

### 2.4. Publication Bias

The funnel plot created using HR and 95% C.I. data will be analyzed to assess selection and publication bias. The extent of systematic publication bias is inversely related to the symmetry of the funnel plot. To adapt the funnel plot for minor insufficient investigations, we may use the standard fail-safe N and Orwin fail-safe N tests. The Begg and Mazumdar ranking correlational analysis will be used to look at the relationship between effect size rankings and their associated variances. To forecast the standardized effects and recalculate the effect size to achieve a symmetrical funnel plot, Egger’s Test of the Intercept and Duval and Tweedie’s trim and fill technique will be utilized.

### 2.5. Dealing with Missing Studies

If any research has missing data, it will only be incorporated in the meta-analysis if the missing data can be obtained by approaching the respective study’s authors. If the data is not accessible, the study will be excluded. Studies with missing or insufficient data will also be excluded from our review.

### 2.6. Risk of Bias

A quality evaluation method for observational and cross-sectional studies has been created by the National Heart, Lung, and Blood Institute. This tool will be used to assess the quality of all the articles that have been chosen. The results of this tool’s analysis will be graded as poor, fair, or excellent [16]. The potential of biases and the quality of the study chosen are inversely related. A high risk of bias denotes poor quality selection, whereas a low risk of bias denotes excellent quality. Any disputes arising during the quality evaluation will be settled by involving a third-party reviewer.

## 3. Statistical Analysis

### 3.1. Meta-Analysis

The heterogeneity between chosen publications will be investigated using meta-regression analysis. The number of patients, year of publication, study duration, research location, kind of study, and diagnostic technique will be investigated for heterogeneity using Cochran’s Q test [42] and Higgins I-squared statistics [43]. Comprehensive Meta-Analysis (CMA) software 3.0 will be used to analyze the Hazard Ratio (HR) with 95% Confidence Intervals (CI). In major investigations, fixed model effects will be employed, and if they are not, random model effects will be used. The overall standard deviation will be calculated using Z-statistics.

### 3.2. Subgroup Analyses

If sufficient studies and recovered data are located and accessible, subgroup analyses will be undertaken based on cervical cancer participants’ clinical, pathological, and biological features, as well as methodological aspects. Our research team intends to look into specific subgroup analyses based on clinical, pathological, and biological data, such as comorbidity, factors associated, tumor histopathology (squamous, adenocarcinoma, clear cell, and undifferentiated), pathological grade (Grade 1, Grade 2, and Grade 3), tumor size, histology grade (well, moderate, poor, undetermined), lymph node metastasis (positive and negative), and vascular connection (positive and negative).

### 3.3. Meta Regression

To identify the source of heterogeneity, a meta-regression analysis will be employed. For heterogeneity, a *p*-value of less than 0.05 is considered significant. The gender distribution, data gathering techniques, sample size, study quality, and sampling strategy will all be examined. By computing R2 with the quantity of the suggested variance, a random-effects model will be chosen and allocated to weigh each research. Meta-regression analysis will be used to explain the heterogeneity of cancer research in relation to one or even more variables of the study, with a large ratio of studies required for reliable regression. For each deviation, a ratio of at least 10 is suggested [44,45,46,47].

### 3.4. Network-Centric Model Analysis

For the same publications stated in this methodology, a pairwise matrix might be created utilizing pairwise correlation or expression correlations of miRNA with patient survival [48,49]. Then, using discrete pattern search algorithms, including cluster-editing and bi-cluster-editing, could be employed so that non-linear pathways/systems might be identified [50,51]. The significance of this methodology relies on the fact that it gives a more accurate and less biased means to identify significant elevated and dysregulated miRNA expression and cervical tumors than other statistical approaches. This is especially the case when the number of factors is larger than five and data reduction techniques such as Principal Component Analysis (PCA) would otherwise be required.

### 3.5. Random Forest Analysis

Random forest analysis is a reliable method for selecting miRNA expression variables. The findings of this study can be utilized to create prognostic value tools such as decision trees. AI prognosis tools might be created by combining random forest analysis with other ensemble methodologies, such as those offered by the R H_2_O package [52]. Randomized forest analysis, on the other hand, is a good technique to find robust features for predictive modeling. PCA and K-means are two other options. These strategies, on the other hand, function best when the number of traits or dimensions is kept under five for most of the variance [53,54].

### 3.6. Heterogeneity Assessment

We will apply the total HR, Cochran’s Q test, and Higgins’ (I^2^) to test the heterogeneity. For the I^2^ test, an I^2^ value of more than 50% suggests heterogeneity. For the Q test, a *p*-value of 0.01% will be considered adequate. On detecting any heterogeneity, a random effect model would be created as well as employed with statistical analysis techniques such as tau-squared statistics, which is used to determine the variance in test accuracy reported in different investigations. Q statistics will also be used to investigate the null hypothesis, and tau squared will be used to determine depression. Forest plots will be created, which will be utilized for in-depth investigation.

## 4. Presenting and Reporting the Review Results

**Figure 1:**
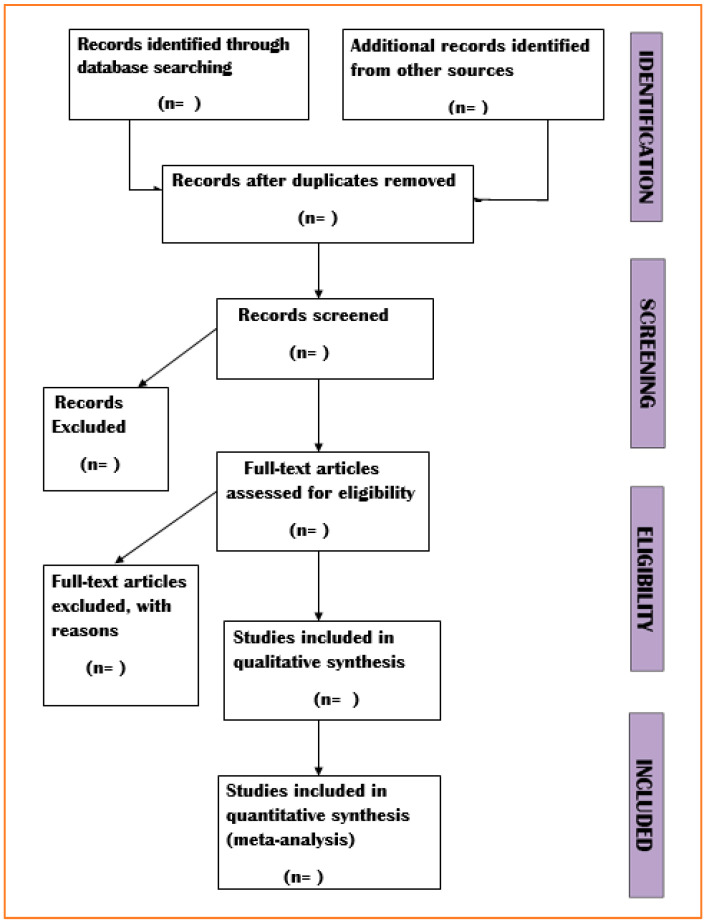
Flow chart: its role has garnered massive attention from researchers who are trying to identify their roles in CC. One of the major tasks carried out by scientists is to identify how well miRNAs regulate chemoresistance in CC. Through this study, we are trying to correlate all the data and increase the understanding of the function of miRNAs in CC.

This protocol will be developed using the PRISMA-P recommendations [55]. The findings will be made public in compliance with PRISMA guidelines [56]. Figure 1 is a flowchart illustrating the screening process (to be utilized). The included studies’ qualitative data will be evaluated descriptively. The outcomes of meta-analyses will be represented as a forest plot. Based on Egger’s graphical test for publication bias, an inverted funnel plot will be used to show publication bias. As a complement, the search strategy, PRISMA-P checklist, and quality evaluation tool will be made accessible.

## 5. Ethics and Dissemination

This analysis derives data from publicly available studies and does not directly involve human participants directly to draw data and, therefore, formal ethical review is not required for this study. The study aims to give a broader view of the role of miRNA in contribution to chemoresistance in cervical cancer. We plan to disseminate the study by publishing it in a peer-reviewed journal and presenting it in relevant conference proceedings. There are no systematic studies or meta-analyses that we are aware of that explore the function of miRNA in chemoresistance in cervical cancer.

## 6. Discussion and Conclusions

The underlying notion of this study is to figure out if miRNAs play a crucial role as a chemoresistance regulator in CC and if they can be used as a marker for identification. Forest plots will be developed using the HR values and 95% C.I. These findings will be utilized to form a final observation. This systematic evaluation and meta-analysis, we feel, will help us better understand the role of miRNA as a chemoresistance regulator in cervical cancer.

HPV is known to be the cause of most CC as indicated by research undertaken in the past few decades, due to their longtime presence. HPV is a common sexually transmitted disease occurring worldwide. However, it can turn cervical tissues malignant when left unattended. Recently, miRNA and its role has garnered massive attention from researchers who are trying to identify their roles in CC. One of the major tasks carried out by scientists is to identify how well miRNAs regulate chemoresistance in CC. Through this study, we are trying to correlate all the data and increase the understanding of the function of miRNAs in CC.

## Figures and Tables

**Table 1:** Keywords used for searching—search strategy.

S.no	Keywords
1.	“Cervical Cancer” (Topic) AND “miRNA” (Topic)
2.	“Cervix Uteri Cancer” AND “miRNAs”
3.	“miRNA as Chemoresistance regulator” (Topic) AND “Cervical Cancer” (Topic)
4.	“Role of miRNA in CC” (Topic)
5.	“CC” (Topic) AND “Drug resistance” (Topic)
6.	“Reduced chemosensitivity by miRNA” (Topic) AND “Cervical cancer” (Topic)

## Data Availability

Not applicable.
